# Update on Peripheral Nerve Electrodes for Closed-Loop Neuroprosthetics

**DOI:** 10.3389/fnins.2018.00350

**Published:** 2018-05-28

**Authors:** Emil H. Rijnbeek, Nick Eleveld, Wouter Olthuis

**Affiliations:** BIOS Lab-on-a-Chip Group, MESA+ Institute for Nanotechnology, MIRA Institute for Biomedical Technology and Technical Medicine, University of Twente, Enschede, Netherlands

**Keywords:** neuroprostheses, peripheral nerve, neural interface, implanted electrode, longevity, spatial resolution

## Abstract

In this paper various types of electrodes for stimulation and recording activity of peripheral nerves for the control of neuroprosthetic limbs are reviewed. First, an overview of interface devices for (feedback-) controlled movement of a prosthetic device is given, after which the focus is on peripheral nervous system (PNS) electrodes. Important electrode properties, i.e., longevity and spatial resolution, are defined based upon the usability for neuroprostheses. The cuff electrode, longitudinal intrafascicular electrodes (LIFE), transverse intrafascicular multichannel electrode (TIME), Utah slanted electrode array (USEA), and the regenerative electrode are discussed and assessed on their longevity and spatial resolution. The cuff electrode seems to be a promising electrode for the control of neuroprostheses in the near future, because it shows the best longevity and good spatial resolution and it has been used on human subjects in multiple studies. The other electrodes may be promising in the future, but further research on their longevity and spatial resolution is needed. A more quantitatively uniform study protocol used for all electrodes would allow for a proper comparison of recording and stimulation performance. For example, the discussed electrodes could be compared in a large *in vivo* study, using one uniform comparison protocol.

## 1. Introduction

Limb amputation is a procedure performed on thousands of patients each year, with lower limb amputation mainly performed in diabetic peripheral neuropathy patients and trauma [incidence of 5.1–200 per 10^5^ population per year (Moxey et al., 2011)] and upper limb amputation mainly after traumatic limb damage [5 per 10^5^ population per year (Winkler, 2009)], immensely affecting the lives of those involved. Due to the technological development of prostheses of the last couple of decades, quality of life can be increased by replacing the (partly) missing limb with a controllable artificial limb. Yet in practice people reject the prostheses over time, mainly because of a lack of sensory feedback, uneasiness of cleaning, prosthesis weight and the absence of independent movement of individual parts of the prosthesis (Pylatiuk et al., 2007). The lack of sensory feedback emphasizes the need for high-quality recording and stimulation electrodes to more natural control of the prosthesis (Biddiss and Chau, 2007). Multiple signals from the body have been used for (feedback-) controlled movement of the prosthesis. The approaches can be roughly subdivided into central nervous system (CNS) based control and peripheral nervous system (PNS) based control of the prosthesis (Warren et al., 2016). Although promising for the future, CNS based approaches for prosthesis control are out of the scope of this review. PNS based control of the prosthesis can be further subdivided into control with electromyographic (EMG) electrodes and electroneurographic (ENG) electrodes.

The main advantage of the EMG-based prosthesis is that it requires non-invasively or minimally invasively obtained EMG-signals as motor input. However, this approach offers a limited number of active degrees of freedom (Ciancio et al., 2016). Furthermore, sensory feedback in any form other than visual feedback is often absent or still non-specific, making it difficult to naturally perform everyday tasks (Sainburg et al., 1995; Johansson and Flanagan, 2009).

ENG electrodes provide selective recording from and stimulation to peripheral nerves. This allows for precise feedback-aided control of a prosthesis, mimicking actual feedback control of muscles in a healthy subject (Ciancio et al., 2016). ENG-electrodes have been successfully implemented clinically in non-neuroprosthetic applications, such as bladder management (Jezernik et al., 2002), drop foot (Liberson, 1961), vagal nerve stimulation (McLachlan, 1997; Fisher and Handforth, 1999) and auditory nerve stimulation (Arts et al., 2003). However, clinical practice of peripheral nerve stimulation for the control of neuroprosthesis is limited (Navarro et al., 2005). The nerve and surrounding tissue may be damaged, because ENG electrodes touch or penetrate the nerve, which limits long term performance. In addition, complex decoding algorithms may be needed to extract the correct information from the noisy electrical signals (Cloutier and Yang, 2013).

Therefore, choosing a suitable peripheral nerve electrode is essential. Many electrodes have been developed for peripheral nerves (Warren et al., 2016) and a comparison of their performance been conducted more than a decade ago (Navarro et al., 2005), but an extensive, quantitative comparison (on e.g., longevity and spatial resolution) of their use in neuroprostheses in recent literature is absent. Recently, Spearman et al. (2017) conducted an extensive review of peripheral nerve interfaces, focusing on electrode design. In the current update, a quantitative comparison of the electrodes is given with the focus on performance. Electrode performance for creating sensory signals and recording motor signals are analyzed separately. The way the electrodes handle mixed signal nerves is out of the scope of this review.

Important electrode requirements are defined and multiple electrode types will be analyzed based upon these requirements. After each electrode is discussed in detail, an overall comparison is made between the electrodes.

### 1.1. Important electrode requirements

Overall electrode usability for neuroprostheses depends on multiple electrode properties. Electrode-tissue interaction is an major group of properties. Important properties in this group are the mechanical mismatch between the tissue and electrode and coating possibilities to influence the immunological reaction of the tissue.

Signal transmission is another class, which is influenced by electrode properties such as impedance and the location of the electrode. In this review however, the different electrodes will be discussed as they are, based on two requirements.

Ciancio et al. summarized multiple requirements for the design of a prosthetic system to re-establish a bidirectional communication with the PNS and foster the prosthesis natural control (Ciancio et al., 2016). Based upon this study, two important requirements were selected for electrode comparison, for which extensive quantitative data is available.

First, it is important to which extent the electrode can be used chronically *in vivo*. This means that the electrode should be able to extract meaningful signals over a long time span. The electrode should inflict little chronic physiological or histological damage due to movement with respect to the surrounding tissue because this can influence the long term performance. Likewise, an inflammatory reaction caused by the electrode material should influence recording or stimulation as little as possible.

Second, maximization of the number of interfaces between electrode and nerve fiber is desired, both for distinguishing different sensory sensations at different areas of the limb and for controlling different parts of the prosthesis. However, placing a large amount of electrodes might be undesirable and therefore a high spatial resolution to limit the amount of electrodes is preferred.

## 2. Electrodes

In this section, various electrodes are discussed based upon the defined requirements. The peripheral nerve electrodes can be divided into three categories, surface electrodes (Cuff electrodes), penetrating electrodes (LIFE, TIME and USEA) and regenerative electrodes.

### 2.1. Cuff electrode

**Figure 1:**
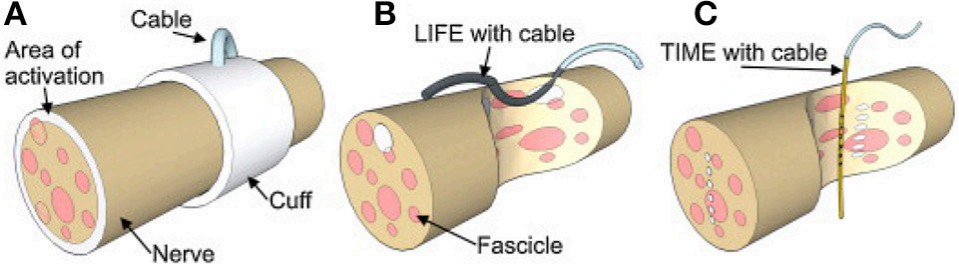
A schematic drawing of **(A)** Cuff electrode, **(B)** LIFE electrode, **(C)** TIME electrode. Figure from Boretius et al. (2010).

**Figure 2:**
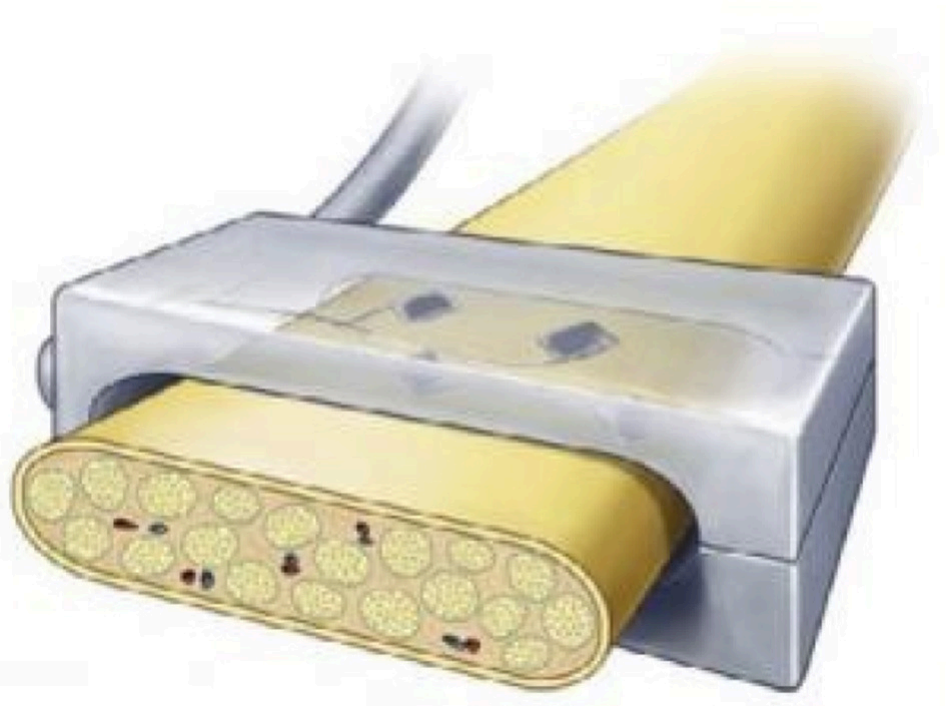
A schematic drawing of a Flat Interface Nerve Electrode (FINE). Figure reproduced with permission from Graczyk et al. (2016).

The cuff electrode is a surface electrode which is wrapped around the nerve (Figure 1A). It measures differences in electrical potential at the outside of the nerve during the propagation of action potentials. There are multiple variants of the cuff electrode. The split ring electrode is a flat ring which has been split at one side such that it can be placed around the nerve (Xue et al., 2015). Naples et al. developed an electrode consisting of conductive segments embedded within a self-curling sheath of bio compatible insulation which gives it a “self-sizing” property (Naples et al., 1988). The other variant is the flat interface nerve electrode (FINE) which flattens the nerve to achieve a greater proximity to the fascicles (Tyler and Durand, 2003) (Figure 2). The FINE is interesting because it gives rise to multiple methods to increase the spatial resolution (Yoo and Durand, 2005; Wodlinger and Durand, 2009).

Depending on the structure of the cuff electrode, silicone (e.g., FINE) and biocompatible polyimides (e.g., split ring electrode) (Dweiri et al., 2017) are often used to shape the cuff and insulate the electrode sites. The electrode sites are often made of platinum based materials (Naples et al., 1988; Dweiri et al., 2017).

#### 2.1.1. Longevity

Cuff electrodes are relatively non-invasive (compared to the penetrating electrodes discussed in the following sections), which positively influences their longevity. Christie et al. showed that a large numbers of cuff electrodes chronically implanted on human peripheral nerves can be stable and work up to 10.4 years (duration of study) (Christie et al., 2017).

FINEs do have an acute effect on nerve functionality as a consequence of mechanical pressure, via changes in nerve myelination and axon density. It was shown, however, that the nerves can recover over time and the electrodes have no further chronic physiological effects (Tyler and Durand, 2003). The nerve can be reshaped significantly without long term physiological or histological damage up to 3 months after implantation (Leventhal et al., 2006).

#### 2.1.2. Spatial resolution

As the cuff does not penetrate the epineurium, it is difficult to achieve highly selective recording from individual fascicles. However, the spatial resolution can be increased by using FINE, which reshapes the nerve and results in the electrodes having a closer proximity to the fascicles.

In addition, the data from multiple electrodes near the nerve can be used to estimate the origin of the signal using various signal processing techniques, like spatial filtering (Wodlinger and Durand, 2009). More recently, a Bayesian Source Filter for signal Extraction (BSFE) algorithm based on spatial filtering was developed (Tang et al., 2014). Wodlinger and Durand identified up to 5 individual fascicles using spatial filtering while recording nerve activity (Wodlinger and Durand, 2011). A study by Tan et al. succeeded in stimulating 10 and 15 unique precept areas on a phantom hand using electrodes implanted for one and two years. They demonstrate that high selectivity and stability can be achieved through an extraneural interface, which can provide sensory feedback to amputees (Tan et al., 2015).

### 2.2. Longitudinal intrafascicular electrode (LIFE)

The longitudinal intrafascicular electrode is a flexible, insulated wire with a small deinsulated region. The wire is surgically inserted into the nerve with a round needle until it reaches a fascicle. It is inserted along the fascicle and then pinched out of the nerve again. The wire is pulled through the insertion until the deinsulated region lays adjacent to the nerve fibers (Malagodi et al., 1989). This is illustrated in Figure 1B.

LIFEs consist of 25–50 μm diameter Pt or Pt-Ir wires insulated with Teflon or metalized Kevlar fibers, mostly insulated with medical-grade silicone. The recording sites are areas of 0.5–1.5 mm long which are left uninsulated (Malagodi et al., 1989; Lawrence et al., 2004).

Lawrence et al. compared Pt-Ir LIFEs and polymer-based Kevlar LIFEs and found that recording characteristics were comparable (Lawrence et al., 2004). Tensile strength and flexibility were best in the multistranded Kevlar LIFEs, which is important for *in situ* long-term recording.

A more recent version of LIFEs is the thin-film LIFEs (tfLIFE), based on a thin micropatterned polyimide substrate. These consist of a highly flexible substrate filament, which can host eight contact sites. Moreover, Thota et al. developed the distributed intrafascicular multi-electrode (DIME), consisting of several (six) LIFEs, which may be used to record from or stimulate even more discrete nerve fiber groups (Thota et al., 2015).

#### 2.2.1. Longevity

The first Pt-Ir LIFEs were quite stiff, which resulted in a relative motion of the electrode within the fascicle. This in turn resulted in a gradual drift of the recorded nerve fiber population and a reduction in signal quality (Goodall et al., 1991). Navarro et al. showed that tfLIFEs functional decline due to surgical implantation and mechanical damage was slight and reversible after 3 months. Moreover, histological evaluation in a rat model after several months showed a mild inflammatory reaction and no evidence of nerve degeneration (Navarro et al., 2007).

#### 2.2.2. Spatial resolution

**Figure 3:**
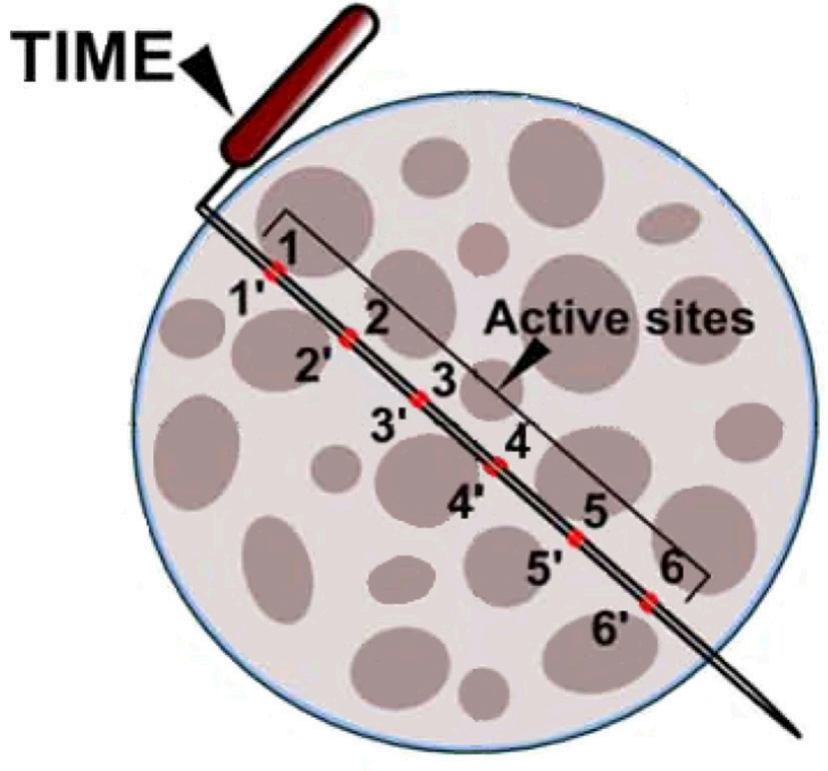
Schematic drawing of a cross section of the median nerve (not to scale), with an implanted TIME electrode. TIME has six equidistant contacts on each side of the polyimide loop. Reproduced with permission from Kundu et al. (2014a).

Even though tfLIFEs electrodes contain 8 individual contact sites, it is still difficult to selectively stimulate or record from individual fascicles with tfLIFE. The electrodes are only in close proximity to part of the fascicles, because of the longitudinal montage of LIFE (Kundu et al., 2014a). This can be seen in Figures 1B, 3. Kundu et al. showed that tfLIFE could selectively activate 2.00 ± 0.89 muscles in a pig animal study (Kundu et al., 2014a).

### 2.3. Transverse intrafascicular multichannel electrode (TIME)

The transverse intrafascicular multichannel electrode (TIME) is developed by Boretius et al. in the international 'TIME-project', funded by the European Union (Boretius et al., 2010). It is designed to be transversally inserted in the nerve. As stated by its developers, it “pursues the objectives of (1) achieving a good contact with nerve fibers, (2) addressing several fascicles over the nerve cross-section to obtain reasonable spatial selectivity and (3) minimizing the mismatch of technical material and nerve tissue.” (Boretius et al., 2010).

**Figure 4:**
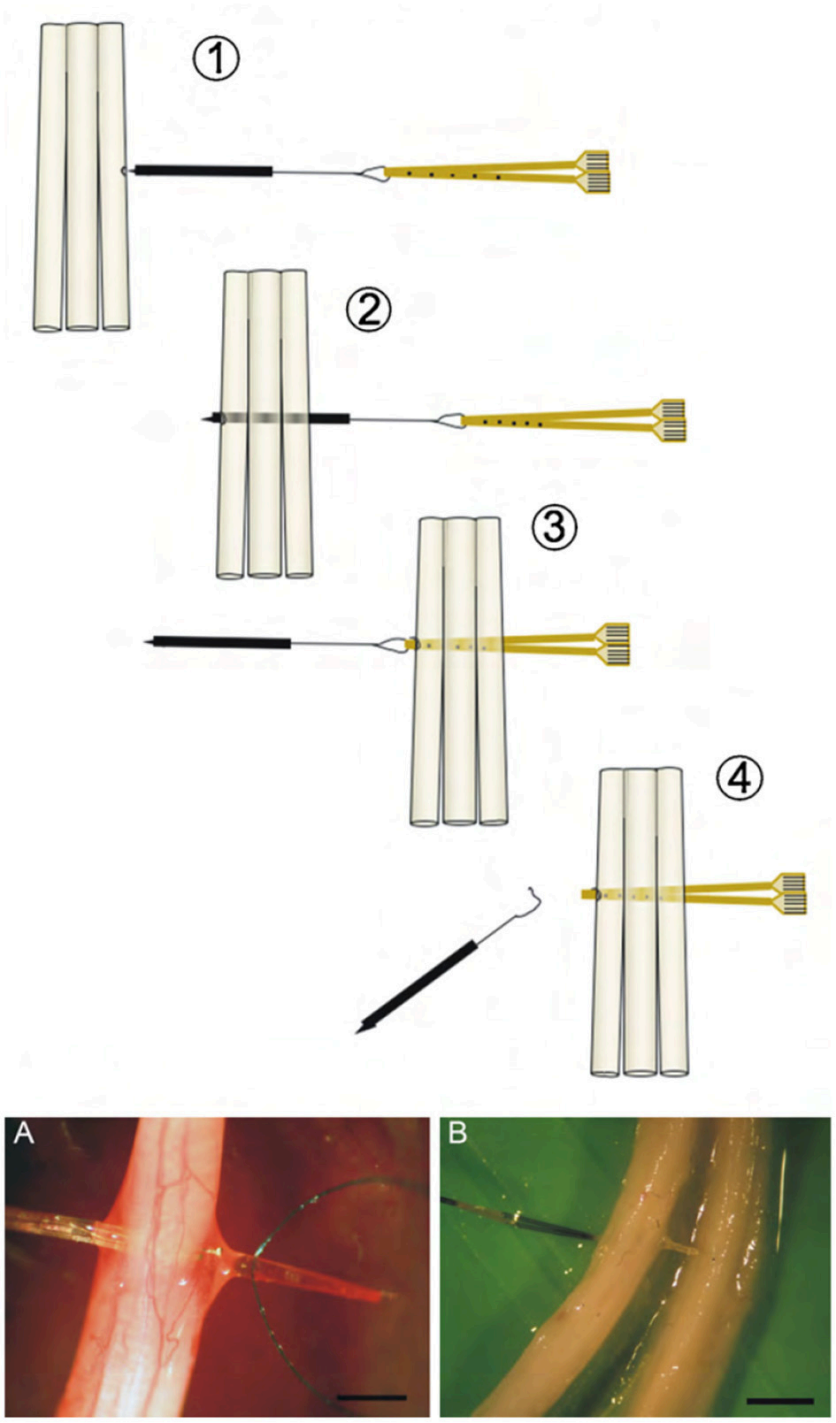
**(Above)** Schematic view of the implementation of the double folded TIME electrode through three fascicles. **(Below)** Photographs of a TIME electrode *in situ*. Scale bar is 5 mm. Reproduced with permission from Boretius et al. (2010).

The TIME-electrode consists of a thin, strip-like polyimide substrate with platinum electrode sites. The substrate is folded to align several electrodes and the folded substrate is threaded transversely through the nerve between the fascicles (Figures 1C, 4). The original design contained 10 electrodes with interelectrode spacing of 230 μm (Boretius et al., 2010). TIME electrodes have already been used in sensory stimulation of the ulnar and median nerve as feedback for the control of a prosthetic hand (Raspopovic et al., 2014).

#### 2.3.1. Longevity

Since one thin device may suffice to interface several groups of nerve fibers, surgical implantation damage is minimized. This may avoid potential nerve damage. Kundu et al. investigated the biocompatibility of TIME on a microscopic scale and they found a layer of fibrosis around the implant but no necrosis or inflammatory cells approximitaly 30 days after implantation (Kundu et al., 2014b).

#### 2.3.2. Spatial resolution

As the TIME is oriented transversely in the nerve (as illustrated in Figure 3), its contact sites lay in close proximity to multiple fibers belonging to different fascicles across the nerve, which should allow for more specific recording and stimulation of individual fascicles than LIFE. Although the TIME was designed for both selective stimulation of and recording from peripheral nerve fascicles, all studies using the TIME have focused on its stimulation characteristics.

Kundu et al. studied the stimulation characteristics of TIME in a pig animal model and were able to selectively activate 3.68 ± 1.49 muscles (Kundu et al., 2014a).

### 2.4. Utah slanted electrode array (USEA)

**Figure 5:**
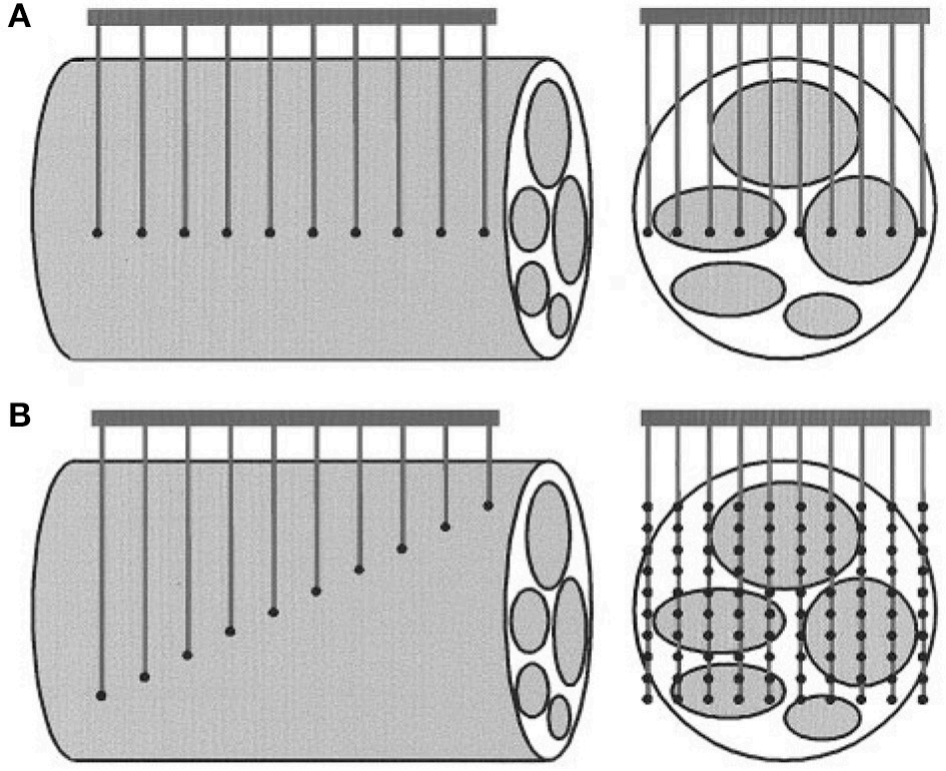
Comparison of the Utah Electrode Array **(A)** and the Utah Slanted Electrode Array **(B)**. Reproduced with permission from Branner et al. (2001).

Another electrode is the Utah Electrode Array (UEA) which consists of a plane with an array of electrodes. An improved version for peripheral nerves is the slanted version (USEA), which is a UEA with electrodes of varying heights, allowing for multiple fascicles at different distances from the electrode to be recorded or stimulated at the same time (Figure 5) (Branner et al., 2001).

The 10-by-10 arrays with 400 μm inter-electrode spacing are often made from a p-doped silicon substrate and are insulated using silicon nitride or glass. The tips of the electrodes are coated using platinum to create a conductive layer (Branner et al., 2001).

#### 2.4.1. Longevity

Multiple studies have evaluated the chronic effects of placement of the USEA, highlighting two main issues. The array needs a large amount of wires to be attached to the electrode, which makes the USEA fragile (Warwick et al., 2003; Branner et al., 2004). In addition, the high amount of penetrating electrodes can cause chronic damage to the nerve due to the movements of the surrounding tissue. Especially the recording performance decreases over time due to connective tissue formation (Branner et al., 2004). These problems could be partly solved by making the device wireless (Sharma et al., 2012). Harrison et al. showed that a wireless neural interface could still accurately transmit recorded potentials in peripheral nerves with a USEA (Harrison et al., 2008). Overall varying results were found on the longevity of this electrode. Some USEAs show no or little chronic damage to the nerve up to 8 weeks (Wark et al., 2014) and 7 months (Branner et al., 2004) after implantation. Others show that there is an inflammatory reaction present after 1 year of implantation (Christensen et al., 2014).

#### 2.4.2. Spatial resolution

High spatial resolution may be expected as multiple fascicles are targeted by the high number of electrodes. Very recently, Davis *et al* implanted USEAs in the median and ulnar nerves of two human subjects with amputated arms. The subjects were able to proportionally control individual fingers of a virtual robotic hand, with as much as 13 different movements after offline decoding and two movements after online decoding (Davis et al., 2016). In addition, stimulation of the individual electrodes evoked multiple percepts that were spatially distributed across the phantom hands in anatomically appropriate distributions. Ledbetter et al. achieved 5 to 10 different muscle contractions in a monkey arm using stimulation via the USEA (Ledbetter et al., 2013).

### 2.5. Regenerative electrodes

**Figure 6:**
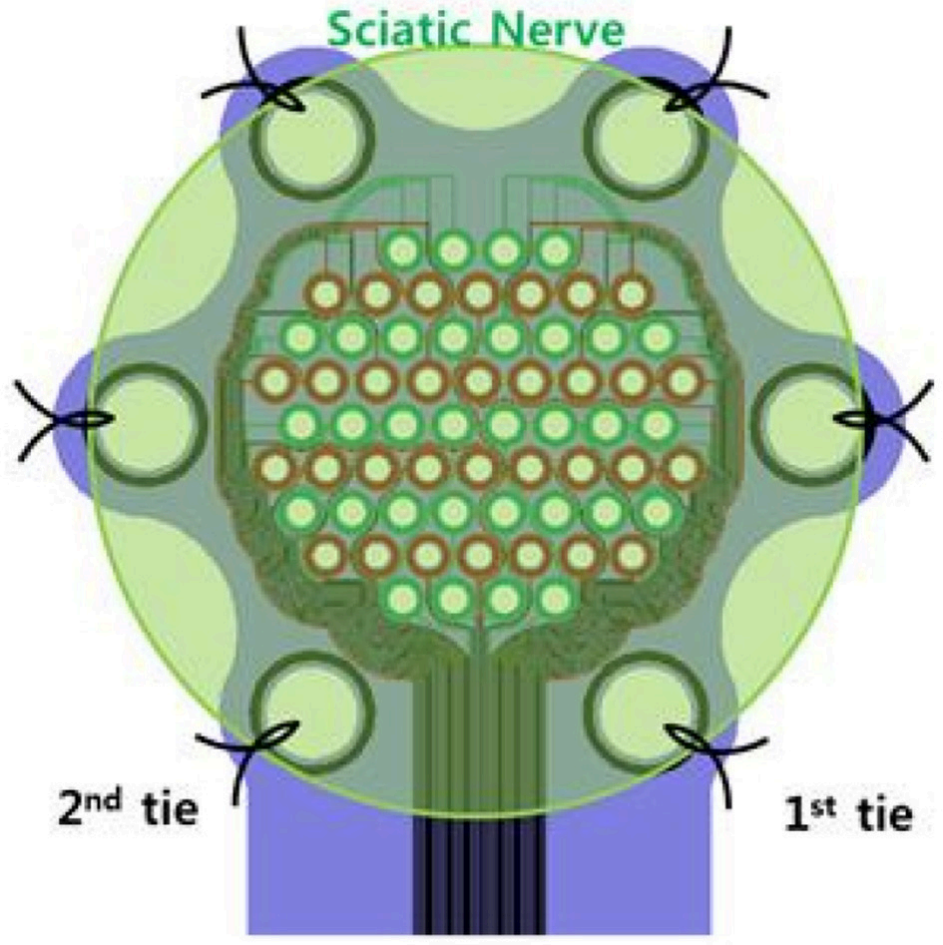
Flat sieve electrode. Reproduced with permission from Jeong et al. (2016).

**Figure 7:**
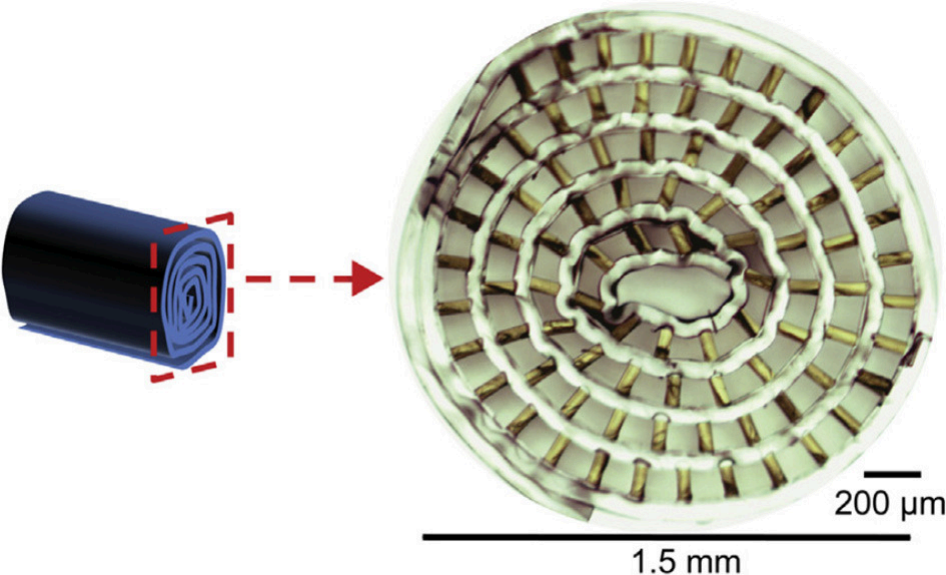
Scaffold sieve electrode. Reproduced with permission from Srinivasan et al. (2015).

The last electrode discussed is the regenerative electrode which uses regeneration to grow the nerve around the electrode instead of puncturing the nerve. Two types of structures can be described, sieve electrodes, and regenerative multi-electrode arrays. The sieve electrode consists of a piece of material with multiple micropores covered with a conductive material and is placed between two ends of a severed nerve. The nerve then regenerates through the pores, after which APs can be evoked or recorded using the conductive material inside the pores (Thompson et al., 2015). Two types of sieve electrodes exist, one is a flat penetrated piece of material (Figure 6), and the other is a scaffold which is folded into a sieve electrode (Figure 7). The regenerative multi-electrode array is designed by having multiple spikes similar to the USEA inside a hollow tube to allow more space and thus faster and non-obstructive regeneration of the nerve (Garde et al., 2009; Seifert et al., 2012). Regenerative electrodes have not yet been used for neuroprothetics and are in experimental phase.

#### 2.5.1. Longevity

This electrode requires severing the nerve before placing the electrode, which has not been necessary for the previously discussed electrodes. The regeneration through the electrode can take 1 week (Garde et al., 2009) to up to a month (Mensinger et al., 2000) and the degree of successful regeneration through the electrodes greatly differs between *in vivo* experiments (Akin et al., 1994; Srinivasan et al., 2015). The fact that the nerve needs to be severed and may not fully recover, makes it the most invasive electrode discussed. However, *in vivo* experiments have been performed in which the fascicles regenerated through the electrode for every implant after which stable long term recording or stimulation (up to 3 months) was possible (Musick et al., 2015; MacEwan et al., 2016). In like manner the regenerative multi-electrode array has shown to provide stable recordings up to 4 months after implantation (Desai et al., 2014). Although no experiments have been performed on human subjects, no evidence has been found for the regenerative electrode causing an inflammatory reaction (Lago et al., 2007).

#### 2.5.2. Spatial resolution

Due to the many templated surface areas in a regenerative electrode, a high spatial resolution is possible. For the sieve electrode the spatial resolution is dependent on the amount of pores made. A small amount of pores makes it possible to create relatively large transit zones, which results in faster regeneration (MacEwan et al., 2016). For a higher selectivity, more pores can be created. It has been shown that sieve electrodes can be created with up to 64 electrodes (Jeong et al., 2016). Although research has been done on individual muscle recording and stimulation, quantitative data on the amount of independently recorded or stimulated muscles is limited. Lago et al. found that it is possible to create at least three individual muscle contractions using the sieve electrode (Lago et al., 2007). The regenerative multi-electrode array was capable of distinguishing 2.9 ± 0.6 individual neurons after 28 days of implantation without any significant change during the following 3 months (Desai et al., 2014). No studies were found in which these electrodes are used for neuroprosthetics.

## 3. Comparison and discussion

Taking all reviewed literature into account, the key characteristics per electrode type are summarized in the following subsections. Furthermore, the signal quality is discussed as a potential criterion.

### 3.1. Longevity

As is shown in Table 1, invasiveness has a negative influence on the long-term functioning of the electrode, resulting from the development of an inflammatory connective tissue layer around the electrode. Only nerve cuffs show stable performance without physiological and histological damage for multiple years. However, newer versions of the invasive electrodes show that the inflammatory response can be limited to a degree that still allows recording and stimulation. This mainly results from higher flexibility, minimizing mechanical stress on the nerve tissue. Longevity studies on non-cuff electrodes, comparable in duration with the cuff electrodes, have not be performed yet. Therefore, the tissue reaction for these electrodes on a timescale of multiple years is not clear yet. Regenerative electrodes may be promising for control of neuroprostheses in the future, but further research into the long term immune response and regeneration of the individual fascicles has to be conducted before they can be used in practice.

**Table 1:** Comparison of the longevity of the discussed electrodes.

**Electrode**	**Longevity**
Cuff	Stable stimulation (after up to 10.4 years) (Christie et al., 2017)
LIFE	Slight and reversible damage (after 3 months) (Navarro et al., 2007)
TIME	Fibrotic layer around the implant, no necrosis or inflammatory cells (after 30 days) (Kundu et al., 2014b)
USEA	Mild or no inflammatory response after 8 weeks (Wark et al., 2014) and 7 months (Branner et al., 2004). Inflammatory reaction after 1 year study (Christensen et al., 2014).
Regenerative	Fascicle regeneration can take up to a month and is not guaranteed. (Mensinger et al., 2000) When regenerated, long-term recording and stimulation may be possible up to 3 (Musick et al., 2015; MacEwan et al., 2016) or 4 months (Desai et al., 2014).

Although it would be better to compare the longevity of the electrodes based on their spatial resolution at many instances (after several weeks/months/years) after implantation, there was not enough available data for all electrodes to make this comparison at the present time.

### 3.2. Spatial resolution

The spatial resolution is the highest for USEAs for recording and highest for cuff electrodes for stimulation, as can be seen in Table 2. The spatial resolution per electrode type differs for stimulation and recording. Most studies only performed a (small scale) quantitative analysis of either recording or stimulation specificity. Furthermore, the quantitative methods used to determine the spatial resolution also varied between the studies, which further complicates a proper comparison of the electrodes. Lastly, the spatial resolution decreases after implantation because of an inflammatory tissue response around the electrode, which can damage the nerve cells and create an insulating layer between the nerve and the electrode (Navarro et al., 2007; Christensen et al., 2014; Kundu et al., 2014b). For example, the USEA may provide specific recording and stimulation characteristics compared to TIME and LIFE, but the inflammatory tissue response may decrease spatial resolution over time.

**Table 2:** Comparison between the spatial resolution of the discussed electrodes.

**Electrode**	**Recording**	**Stimulation # individual muscles**
Cuff	Up to five fascicles (Spatial Filtering) (Wodlinger and Durand, 2011)	Up to 10 and 15 percept areas triggered (spiral cuff) (Tan et al., 2015).
LIFE	No quantitative data available	2.00 ± 0.89 (Kundu et al., 2014a)
TIME	No quantitative data available	3.68 ± 1.49 (Kundu et al., 2014a)
USEA	13 different movements (Offline Decoding) (Davis et al., 2016)	5 to 10 (Ledbetter et al., 2013)
Regenerative	Due to high number of holes, high specificity may be possible	At least three (Lago et al., 2007; Desai et al., 2014)

### 3.3. Signal quality

Besides the other requirements, signal quality is also important. If a signal is too unstable or has too much noise, it will not be suitable for neuroprosthetics. One way to describe the signal quality is the signal-to-noise ratio as has been done by multiple studies (Branner and Normann, 2000; Yoo and Durand, 2005; Citi et al., 2011; Srinivasan et al., 2015; Dweiri et al., 2017; Lee et al., 2017). The problem with SNR is that the definition is not consistent because the bandwidth and the signal content of the measured signal is often not defined. Most of these studies define SNR as the ratio between the amplitude of the signal peak and the root mean square of the noise. But even with this definition, it is hard to compare the electrodes due to the difference in impedance, spatial resolution (Navarro et al., 2005) and inconsistent SNR results across studies published so far.

## 4. Conclusion

Multiple peripheral nerve electrodes for the feedback-aided control of neuroprosthetic limbs have been compared. Important properties for electrodes for neuroprostheses are longevity and spatial resolution for stimulation and recording. The cuff electrode seems to be a promising electrode for the control of neuroprostheses in the near future, because it shows the best longevity and good spatial resolution. Furthermore, it has been used on human subjects in multiple studies. The longevity of TIME and LIFE electrodes on the time-scale of years has not been researched yet, which prevents clinical usage. The USEA shows a high spatial resolution, but its longevity is not high enough yet to be used clinically for neuroprostheses.

The regenerative electrode shows much promise on both spatial resolution and longevity. Although the regenerative electrode requires severing of the nerve, this may not be a problem for patients with an already amputated limb. This may make the regenerative electrode very useful for neuroprosthetic control. Nonetheless, a lot of research needs to be done before it can be effectively used in practice.

In general, a more quantitatively comparable study protocol across multiple research groups is necessary to properly compare the stimulation and recording properties of the different electrode types. For example, the discussed electrodes could be compared in a large *in vivo* study, using one uniform comparison protocol.

## Author contributions

NE and ER are the first authors of the manuscript. They contributed equally to performing literature research and writing the manuscript. WO provided in the scientific guidance from the BIOS Lab-on-a-Chip group and performed thorough proof-reading before submission of the manuscript.

### Conflict of interest statement

The authors declare that the research was conducted in the absence of any commercial or financial relationships that could be construed as a potential conflict of interest.
